# Who Is the Surgeon Now: Human Hands or Machine Minds? Artificial Intelligence in Orthopedics from Diagnosis to Follow-Up—A Structured Narrative Review

**DOI:** 10.3390/jcm15062165

**Published:** 2026-03-12

**Authors:** Furkan Yapıcı

**Affiliations:** Department of Orthopedics and Traumatology, Erzincan Binali Yıldırım University, 24100 Erzincan, Turkey; furkanyapici@hotmail.com

**Keywords:** artificial intelligence (AI), machine learning (ML), deep learning (DL), orthopedics, musculoskeletal imaging, fracture detection, perioperative risk prediction, radiomics, natural language processing (NLP), generative AI (GAI)

## Abstract

**Background:** Artificial intelligence (AI) is transitioning from proof-of-concept prototypes to clinically utilized tools in orthopedics. The key translational question is whether AI will replace surgeons or, more realistically, augment human expertise. **Methods:** A structured narrative review was conducted using PubMed/MEDLINE, Web of Science, and Google Scholar (completed 31 January 2026). Peer-reviewed English-language studies that utilized AI for orthopedic clinical problems were eligible. To synthesize the 73 included papers without forced quantitative pooling, evidence was qualitatively charted and organized using a four-axis framework: clinical task, data modality, validation maturity, and intended user/setting. **Results:** The evidence base was dominated by retrospective, imaging-centered AI studies (predominantly LOE III). Radiograph-based fracture detection and automated measurements were frequently reported to achieve high discrimination, though performance degraded in complex or “edge” cases. Predictive models for arthroplasty and spine outcomes demonstrated variable actionability and inconsistent reporting of calibration. Common translational barriers across subspecialties included limited external validation, dataset shift, and a scarcity of prospective impact studies. **Conclusions:** Current evidence supports an augmentation paradigm rather than a replacement paradigm. AI acts as a “co-surgeon,” improving triage and standardizing quantification. However, safe clinical translation requires representative external validation, rigorous failure analysis, and human-in-the-loop workflows where surgeons retain ultimate accountability.

## 1. Introduction

Artificial intelligence (AI) has evolved from proof-of-concept prototypes to widely implemented clinical tools in orthopedics, especially in image interpretation, perioperative risk prediction, and workflow decision support. Recent orthopedic-focused reviews consistently characterize an expanding “AI ecosystem” that influences nearly every stage of care, from initial diagnosis to rehabilitation and follow-up [1]. Pathway-focused studies additionally emphasize the application of AI across the continuum of care, including diagnosis, treatment selection, surgical support, and outcome monitoring [2].

However, the central debate goes beyond whether AI can “read X-Rays” or “predict complications.” The critical question is whether AI can replace the orthopedic surgeon, or whether its most realistic role is augmentation: supplying faster, more consistent pattern recognition and prediction while retaining human accountability, values, and procedural expertise. In orthopedics, in which decision-making is multimodal—incorporating imaging, clinical examination, intraoperative findings, and patient goals—and errors may result in permanent disability, the “replacement versus augmentation” issue is fundamental. This distinction influences the interpretation of evidence, the selection of validation standards, and the definition of “clinically deployable” AI.

Accordingly, this review is structured around the clinically relevant question: “Who is the Surgeon Now: Human Hands or Machine Minds?” It examines areas where AI is functionally useful, where it is still at the research stage, and the barriers that must be addressed for safe clinical translation.

### 1.1. Why Orthopedics Is an Ideal “AI Testbed”

Orthopedics is highly multimodal, relying heavily on standardized imaging (radiographs, CT, MRI) for diagnosis, classification, and treatment selection. This results in large volumes of structured data, providing an ideal substrate for supervised learning. Consequently, the specialty has seen rapid AI development across diverse domains, including fracture detection in trauma, automated structural segmentation in sports medicine and degenerative diseases, radiomics for musculoskeletal oncology, and predictive modeling for perioperative outcomes in arthroplasty and spine surgery. Because orthopedics demands both high-volume rapid triage and complex, high-stakes surgical planning, it serves as an excellent clinical testbed for evaluating the real-world utility of AI [3,4,5,6,7,8,9,10,11,12,13,14,15,16,17].

### 1.2. What Is Meant by “AI” in This Review

In orthopedic literature, “AI” frequently serves as an umbrella term. For the purposes of this review, AI refers to computational methods that learn patterns from data to support perception, measurement, prediction, and decision-making in medical workflows. This review distinguishes between traditional machine learning, which generally relies on engineered features and classical estimators (such as logistic regression, random forests, and gradient-boosting), and deep learning, which learns hierarchical representations directly from raw inputs and is most commonly implemented using convolutional neural networks for imaging [18].

Across subspecialties, most orthopedic AI studies fall into recurring clinical task families. These include detection and classification (for example, fracture presence or tear type), segmentation and quantification (for example, delineating cartilage or measuring deformity), prediction and prognostication (for example, complications, readmission, or functional outcomes), and decision support (for example, triage prioritization or pathway guidance). This task-based framing is consistent with prior methodological syntheses and helps separate “high-accuracy” perception tasks from more clinically fragile prognostic models [19].

Radiomics is considered separately from end-to-end deep learning because it converts images into predefined quantitative features (shape, intensity, texture) that are subsequently modeled with ML. In orthopedics, radiomics has been used most often in oncology and degenerative imaging, where texture heterogeneity and multi-sequence information may correlate with biology, response, or prognosis.

Finally, orthopedic AI increasingly includes text-based and generative systems. Natural language processing is used to extract clinically meaningful signals from notes, operative reports, and discharge summaries, and it has been incorporated into readmission prediction and automated complication identification [15]. Large language models and other generative AI tools have also been proposed for documentation support, evidence summarization, patient communication, and coding assistance, but they bring new governance concerns (e.g., hallucination, provenance, privacy) distinct from those in classic supervised prediction [20].

### 1.3. The Central Question: Replacement or Augmentation?

The concept of “replacement” is an inappropriate default benchmark. Orthopedic surgery is not a single cognitive task but rather a workflow comprising coupled decisions and actions: synthesizing patient history, examination, imaging, and preferences; selecting a treatment strategy in the face of uncertainty; executing technically demanding procedures; managing complications and rehabilitation; and communicating risks, trade-offs, and goals.

To reduce rhetorical ambiguity, we must establish an operational definition for these concepts. In this review, an evidentiary threshold for true “replacement” is defined as an AI system capable of autonomous clinical decision-making and execution without human oversight or direct medico-legal accountability. Achieving this would require models to prospectively demonstrate not only superior diagnostic accuracy across unrestricted populations but also the capacity to safely manage therapeutic interventions and unexpected complications. Conversely, “augmentation” is operationally defined as human-in-the-loop intelligence. In an augmentation framework, AI provides high-speed, probabilistic assistance (e.g., flagging anomalies, automating standard measurements, or stratifying risk), while the clinician synthesizes this data with patient values to retain ultimate decision-making authority and accountability.

AI can exceed human performance in narrowly defined tasks, such as binary detection under standardized conditions. However, “replacement” would require effective performance across the entire spectrum of clinical responsibilities, including accountability and moral reasoning.

A more realistic target is human-in-the-loop intelligence.

Most clinically plausible orthopedic AI use cases are augmentative, including triage support (flagging critical findings), second-reader assistance (reducing missed diagnoses), measurement automation (segmentation-driven quantification), risk stratification (complications and readmissions), and workflow optimization.

Subspecialty reviews increasingly frame AI as a decision-support tool rather than a substitute for the clinician, notably in complex MRI interpretation domains such as the shoulder [13].

Methodological reality check: bias, generalizability, and confounding. The “can AI replace the surgeon?” question often collapses into a more practical one: can AI be trusted outside the dataset it was trained on? A widely cited warning comes from work showing that deep learning can appear to predict hip fractures using confounding patient and healthcare system variables—highlighting that high AUC does not necessarily mean clinically meaningful reasoning [21].

This is directly relevant to orthopedics because many models are trained on retrospective data with hidden shortcuts (scanner/site signatures, projection artifacts, annotation methods, implant prevalence, demographic skews). Absent robust external validation, calibration, and (ideally) prospective impact studies, “replacement” is not just premature—it is conceptually not aligned with how AI performance is often achieved.

Generative AI intensifies the accountability problem. LLMs and generative systems introduce additional failure modes: incorrect yet certain outputs, provenance ambiguity, and privacy leak risks. Even if GAI accelerates documentation and education, clinical decision support using generative models raises higher evidentiary and governance thresholds than classic image classifiers [20].

The working position of this review is that, in the near- to mid-term, the dominant trajectory is augmentation rather than replacement. In this model, the surgeon remains the accountable decision-maker, and AI serves as a high-speed, probabilistic assistant.

### 1.4. Scope, Aims, and Narrative Review Questions

This review follows AI applications across the orthopedic care continuum, from diagnosis and triage using radiographs, CT, MRI, and ultrasound to preoperative planning and risk prediction, intraoperative support (including navigation and robotics interfaces where studied), postoperative outcome and complication prediction, and follow-up and rehabilitation (monitoring and patient engagement tools). This end-to-end structure replicates pathway-oriented syntheses that track orthopedic care from diagnosis through rehabilitation [2].

The aims of this review are to map the roles assigned to AI in orthopedics across various tasks and data modalities; to appraise the maturity of the evidence base with particular attention to external validation, calibration, and impact evaluation; to distinguish use cases that are plausibly deployable from those that remain research-grade; and to critically examine the replacement narrative by evaluating accountability, bias, and translational barriers.

Accordingly, the Results and Discussion address where AI matches or exceeds clinician performance and under what conditions; which workflows benefit most from human-in-the-loop assistance versus automation; which methodological pitfalls most commonly limit generalization and clinical impact; and how orthopedic teams can evaluate, govern, and integrate AI tools safely.

### 1.5. Conceptual Framework for the Paper

To keep ‘AI in orthopedics’ clinically interpretable, the included studies were organized using a pragmatic four-axis framework. Specifically, each application was described by clinical task (detection, segmentation, classification, prediction, or decision support), data modality (imaging, tabular clinical data, text/NLP, or multimodal combinations), validation stage (internal, temporal, external, and/or prospective or impact evaluation), and intended user and setting (radiologist, orthopedic surgeon, emergency clinician, or rehabilitation team; academic vs. community practice; elective vs. emergency care).

## 2. Materials and Methods

### 2.1. Review Design and Scope

This study was designed as a structured narrative review to synthesize clinical and translational evidence on artificial intelligence (AI) in orthopedics across the continuum of care. While the manuscript incorporates structured elements to enhance transparency—such as a search strategy, eligibility criteria, and a PRISMA-style flow diagram—it does not seek to draw systematic inferences. Because the evidence base spans fundamentally different analytic designs (e.g., imaging algorithm development, registry-based prognostic modeling, natural language processing, and narrative syntheses), applying a single formal risk-of-bias (RoB) tool or AI-specific quality frameworks (such as TRIPOD-AI, PROBAST, CLAIM, or RQS) uniformly across all studies was deemed methodologically inappropriate. Instead, a descriptive, structured narrative approach was chosen to facilitate interpretation across subspecialties, data modalities, and validation stages without imposing forced quantitative pooling.

### 2.2. Search Period and Information Sources

The literature search process was initiated on 1 January 2026 and completed on 31 January 2026 (last search date: 31 January 2026). Three databases were used to maximize capture across biomedical, engineering-adjacent, and interdisciplinary orthopedic AI literature: PubMed/MEDLINE, Web of Science (Core Collection), and Google Scholar.

To identify influential or highly cited studies that may not be consistently indexed or retrieved consistently across platforms, reference list screening and citation chasing of key included papers were also performed.

### 2.3. Search Strategy and Keywords

A concept-combination search strategy was built around two primary term blocks:

Orthopedics/musculoskeletal block: orthopedics/orthopedics, musculoskeletal, fracture, arthroplasty, spine, scoliosis, sports medicine, soft-tissue pathology (e.g., meniscus, rotator cuff), and musculoskeletal oncology terms (e.g., osteosarcoma).

AI methods block: artificial intelligence, machine learning, deep learning, radiomics, natural language processing (NLP), and large language models/generative AI.

Representative database-specific search strings were as follows:

***PubMed***

(orthopedics OR orthopedic* OR orthopedic* OR musculoskeletal OR fracture* OR arthroplasty* OR spine OR scoliosis OR meniscus OR “rotator cuff” OR osteosarcoma) AND (“artificial intelligence” OR “machine learning” OR “deep learning” OR radiomics OR “natural language processing” OR NLP OR “large language model*” OR “generative AI”)

***Web of Science***

TS = (orthopedics* OR musculoskeletal OR fracture* OR arthroplasty* OR spine OR scoliosis OR meniscus OR “rotator cuff” OR osteosarcoma)

AND TS = (“artificial intelligence” OR “machine learning” OR “deep learning” OR radiomics OR “natural language processing” OR NLP OR “large language model*” OR “generative AI”)

***Google Scholar***

(“artificial intelligence” OR “machine learning” OR “deep learning” OR radiomics OR “natural language processing” OR “large language model” OR “generative AI”)

orthopedics OR orthopedics OR musculoskeletal OR fracture OR arthroplasty OR spine OR scoliosis OR meniscus OR “rotator cuff” OR osteosarcoma

In Google Scholar, additional targeted keyword combinations were used when needed to improve retrieval for specific clinical tasks (e.g., “fracture detection radiographs deep learning”, “arthroplasty complication prediction machine learning”, “Cobb angle deep learning”, “osteosarcoma radiomics MRI”), and citation counts were used pragmatically to rank highly influential records during screening when multiple near-duplicate or highly similar entries were encountered.

Google Scholar was used as a supplementary source to capture interdisciplinary studies and early online publications that may not yet be fully indexed in PubMed/MEDLINE or Web of Science. To improve reproducibility despite Google Scholar’s dynamic ranking, screening was restricted to the first 200 results sorted by relevance. Duplicate and near-duplicate records were resolved using DOI/PMID and title/author matching. Citation counts were not used as inclusion criteria.

### 2.4. Eligibility Criteria

Inclusion criteria. Records were eligible if they met all of the following:

1. Addressed an orthopedic or musculoskeletal clinical problem where AI was a primary analytic component;
2. Reported an AI application aligned with at least one of the review’s task families (detection/classification; segmentation/measurement; prediction; decision support); and
3. Were available as full-text, peer-reviewed manuscripts in English.
4. Original studies (development and validation), clinical evaluation/reader-impact studies (when available), systematic reviews/meta-analyses, and high-yield narrative syntheses that materially informed clinical interpretation were included.
5. Conference abstracts without full text, non-musculoskeletal applications, and papers in which AI was not a substantive analytic component (e.g., passing mentions without model development/validation or clinically interpretable AI results) were excluded.

### 2.5. Study Selection and Evidence Set

Search results were deduplicated prior to screening. Titles and abstracts were screened for relevance, and full texts were reviewed when necessary to confirm eligibility and extract methodological details. Reference list screening and citation chasing were used iteratively to identify further pertinent studies that supported the review’s scope.

Through this process, the final narrative evidence base consisted of 73 included papers.

### 2.6. Data Charting and Extracted Variables

From each included study, data were charted to support clinically interpretable synthesis across heterogeneous AI applications. Extracted items included:

- publication details (author, year, journal),
- orthopedic subspecialty/domain,
- clinical task and intended clinical decision point (triage/diagnosis; planning; perioperative risk; follow-up),
- data modality (imaging, tabular clinical data, text/NLP, or multimodal),
- AI approach category (traditional ML, deep learning, radiomics, NLP, or LLM/generative AI, where applicable),
- reference standard (e.g., expert labels, CT/MRI confirmation, arthroscopy, pathology, longitudinal outcomes), and
- validation strategy (internal, temporal, external, and/or reader/workflow impact evaluation when reported).

### 2.7. Evidence Synthesis

Given substantial heterogeneity across datasets, outcomes, validation strategies, and outcome metrics, findings were synthesized using a structured qualitative narrative rather than a meta-analysis. Evidence was summarized by subspecialty and cross-cutting task category and interpreted alongside translational maturity signals such as external validation, calibration reporting for prognostic models, and reader/workflow impact studies.

### 2.8. Risk of Bias (RoB) and Level of Evidence (LOE) Analysis

Given that this study is a narrative review encompassing multiple methodological families (including imaging algorithm development, registry or EHR-based prediction modeling, NLP pipelines, and evidence syntheses), a single formal risk-of-bias (RoB) tool was not applied to the entire evidence base. Such tools are not uniformly relevant across fundamentally different analytic designs and may yield misleading comparability when used as a universal scoring system. Therefore, the methodological appraisal strategy was limited to a design-based Level of Evidence (LOE) classification, intended to provide a transparent overview of the evidentiary structure of the included literature.

We acknowledge the inherent limitations of applying traditional surgical LOE hierarchies to AI research. Conventional LOE frameworks are not well-aligned with retrospective model development or registry-based prediction modeling, as they do not adequately capture critical AI-specific methodological vulnerabilities, including overfitting, data leakage, and calibration failure. In this review, AI model development papers were mapped onto conventional LOE categories primarily based on their data source and validation design (e.g., retrospective dataset validation was mapped to LOE III). Notably, our final evidence base contained no LOE IV studies; this absence reflects the current paradigm of AI development, which relies on large-scale retrospective datasets (LOE III) rather than small clinical case series (LOE IV). Consequently, LOE is utilized here strictly as an indicator of study design rather than a comprehensive measure of algorithmic internal validity or clinical readiness.

### 2.9. Ethics and Data Availability

Ethics approval was not required, as this study relied exclusively on published literature. All synthesized data were obtained from publicly available sources and are cited in the manuscript reference list. The Grammarly app (Version 1.2.239.1849) was used for language editing in this study.

### 2.10. Yield

**Figure 1:**
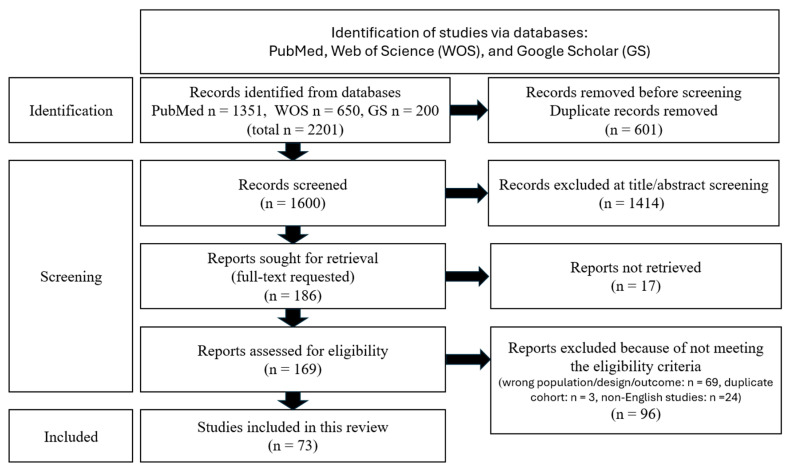
PRISMA-style flow diagram.

A PRISMA-style flow diagram is presented as Figure 1. Across the 73 included papers, the LOE distribution was: LOE I: 7/73 (9.59%), LOE II: 4/73 (5.48%), LOE III: 48/73 (65.75%), LOE IV: 0/73 (0.00%), LOE V: 13/73 (17.81%), and unclassifiable: 1/73 (1.37%).

## 3. Results

To provide a structured overview of the clinical maturity and evidence landscape across different orthopedic subspecialties, the 53 original AI development and validation studies included in this review are summarized in Table 1. The remaining 20 references in our evidence base [1,2,3,4,11,12,13,14,15,18,19,20,22,23,24,25,26,27,28,29] consist of systematic reviews, meta-analyses, and narrative frameworks. Because these 20 articles synthesize prior literature rather than train or validate novel AI models, they do not possess a primary “Validation Stage” or “Data Modality” of their own. Consequently, they are excluded from the model-centric summary in Table 1, though their conceptual findings are integrated throughout the qualitative synthesis below.

### 3.1. Overview of the Included Literature (All Subspecialties)

Across the included papers, the literature was dominated by imaging-centered AI (radiographs, MRI, CT), with a smaller but clinically important group using tabular perioperative/registry variables and clinical text (NLP). Based on the distribution of topics within the included evidence base, trauma/fracture care accounted for the largest share, followed by sports/soft-tissue and OA imaging, arthroplasty, spine, and a focused set of musculoskeletal oncology radiomics/ML studies.

A recurring pattern across subspecialties was a mismatch between very high technical performance in selected datasets and the more modest, heterogeneous gains seen when models are externally validated, tested on “difficult” edge cases (e.g., subtle/occult fractures; extreme scoliosis), or evaluated for clinical utility (reader studies, calibration, net benefit, workflow endpoints).

#### 3.1.1. Study Types and Clinical Preparedness

Study types (as represented in this evidence base). The evidence base comprises numerous original development/retrospective validation studies, as well as a substantial body of systematic reviews/meta-analyses and narrative reviews that consolidate the field.

A smaller (but highly relevant) segment comprises reader/impact studies that demonstrate how AI affects clinician performance—these are especially useful for assessing “deployability” rather than depending solely on AUROC.

Examples of “clinical preparedness” signals. Multi-site performance + broad anatomy coverage: A multi-site evaluation showed strong overall discrimination for fracture detection throughout adult MSK radiographs (AUC 0.974, sensitivity 95.2%), with variable performance by region (e.g., lower performance in the foot). This sort of broad, multi-anatomic, multi-site evidence moves closer to “real-world readiness” [30].

Human–AI teaming in controlled reader settings: An observer study demonstrated that AI assistance improved clinician diagnostic performance (AUC rising from 0.90 to 0.94; sensitivity 82%→90%; specificity 89%→92%) and narrowed gaps between clinician groups. This study design is highly relevant to implementation [31].

External validation (geographic + dataset shift): Multi-continental external validation remained explicitly used in ACL tear detection work, where AUC remained high (~0.94) and retraining on external datasets further improved performance in some cohorts [65].

Calibration + decision-curve analysis (beyond AUC): In arthroplasty and spine outcome prediction, several studies reported not only discrimination but also calibration and decision-curve net benefit, which are key for risk models intended for shared decision-making and perioperative planning [48,56].

Where readiness is still limited. Many studies remain single-center, retrospective, and/or lack temporal/external validation, consistent with limitations repeatedly highlighted in subspecialty reviews (e.g., arthroplasty and shoulder MRI) [13,14].

While discrimination metrics (e.g., AUROC) were ubiquitously reported, markers of translational maturity were substantially less common. Among the 73 included studies, explicit external validation across geographically distinct cohorts or different institutions was reported in only 12 studies (16%). Furthermore, despite the recognized importance of clinical utility in prognostic modeling, formal calibration assessment was reported in just six studies (8%), and decision-curve analysis (DCA) to evaluate net clinical benefit was utilized in only four studies (5%). Prospective impact evaluations or real-world deployment data were notably rare, appearing in three studies (4%). This quantification underscores that while the technical feasibility of orthopedic AI is well-established, rigorous translational validation remains the exception rather than the rule.

The vast majority of the remaining original studies (n = 41, 56%) were strictly constrained to internal validation (e.g., random train-test splits from a single institution) and relied exclusively on basic discrimination metrics, while the rest of the evidence base comprised systematic and narrative reviews (n = 20, 27%) that synthesized these early-stage findings.

#### 3.1.2. Data Modalities and Gold Standards

Dominant modalities are: Radiographs for fracture detection, triage prioritization, pediatric fracture detection, and measurement tasks (e.g., Cobb angle; fracture morphometrics) [4,32]; MRI for sports/soft-tissue diagnosis (meniscus, ACL, rotator cuff), cartilage segmentation, and oncology radiomics [10,11]; CT for radiomics in oncology; fracture-risk prediction; selected prediction workflows [33,72]; tabular perioperative/registry/EHR data for arthroplasty complication prediction, DVT risk, discharge disposition, spine outcomes [49,57]; Text/NLP for readmission prediction and operative-report mining for complications [15,50]; ground truth patterns. Expert labeling (radiologists/orthopedists) remains common but causes variability and label noise; higher-quality ground truths included for CT/MRI confirmation for “occult” fractures or subtle injury [6]; arthroscopy for meniscus/ACL ground truth (when available across included studies) [11]; pathology endpoints (e.g., necrosis response) for osteosarcoma radiomics [22].

#### 3.1.3. Common AI Tasks Across Orthopedics

Across subspecialties, studies concentrated on a small set of recurring task families:

- **Detection/classification:** Fracture detection and triage on radiographs (multi-anatomic) [30]; ACL tear detection on MRI with external validation [65]; Rotator cuff tear screening on multi-plane MRI [66].
- **Segmentation:** Knee cartilage and structures for automated morphometrics in OA cohorts [10].
- **Measurement automation:** Cobb angle measurement automation (scoliosis) and fracture feature quantification [34,51].
- **Outcome prediction/risk stratification:** Arthroplasty complications, DVT, infection, discharge disposition; spine surgery outcomes; metastatic spinal tumor neurologic recovery [52,56,57].
- **Decision support/workflow impact:** Reader assistance studies that quantify effects on AUC, sensitivity, and specificity, especially among non-expert readers [31,32].

#### 3.1.4. Common Failure Modes Reported in the Literature

Several recurrent failure modes limit translation across subspecialties. First, confounding and shortcut learning can inflate performance when models exploit patient, process-of-care, or acquisition artifacts rather than clinically meaningful signals, as illustrated by hip fracture prediction, which collapses toward chance when confounders are balanced [21]. Second, domain shift and gaps in external validation mean that high internal discrimination may not transfer across institutions, devices, or populations, leading to missed adaptation, even in settings where multi-continental validation has demonstrated feasibility [65]. Third, class imbalance and rare-event prediction can yield only moderate discrimination and unstable thresholds, so calibration and impact evaluation become essential for low-base-rate endpoints, such as some arthroplasty complications [58,59]. Finally, methodological and reporting deficits, particularly in radiomics, threaten repeatability and should be addressed by transparent reporting and validation practices [23].

### 3.2. Trauma and Fracture Care

#### 3.2.1. Diagnostic Triage and Fracture Detection on Radiographs

Performance in broad fracture detection. A multi-site deep learning system showed very high overall discrimination for adult MSK fracture detection (AUC 0.974) with high sensitivity (95.2%), yet notable regional variability (e.g., the foot had the lowest AUC). This supports the feasibility of ED triage but also points out that “overall AUC” can mask weak anatomical subdomains [30].

Clinical effect: AI assistance improves human performance. In a reader-assistance design, fracture detection improved when clinicians were aided by AI (AUC 0.90→0.94; sensitivity 82%→90%; specificity 89%→92%). This is a key “translation-ready” outcome because it quantifies benefit in a human workflow rather than only model-vs-label accuracy [31].

Hip fracture detection is a high-impact target because delayed recognition has major downstream consequences. Both early development studies and subsequent larger evaluations have assessed deep-learning systems for detecting and visualizing hip fractures on pelvic radiographs, including reader-performance designs evaluating the effect of AI assistance on radiologist accuracy and efficiency [7,8,35].

Foot/ankle as an “edge-case stress test”. In ankle fracture detection, CNN performance was reported to be extremely high with a multi-view strategy (sensitivity 98.7%, specificity 98.6%), underscoring that multi-view input can meaningfully stabilize performance when single-view ambiguity is common [36].

Synthesis in reviews. A dedicated literature review on radiograph fracture detection in the evidence base consolidates advances in performance, clinical use cases, and common limitations (e.g., dataset bias, scarcity of external validation) [4].

#### 3.2.2. Subtle/Occult Fractures and “Missed Injury” Scenarios

Scaphoid fracture as a model problem. A deep learning pipeline for scaphoid fractures achieved strong performance for apparent fracture detection (AUROC ~0.955, sensitivity 87.1%) but notably lower performance for occult fracture identification (AUROC ~0.810, sensitivity 79.0%, specificity 71.6%), indicating the intrinsic difficulty and the “spectrum” from obvious to subtle injuries [5].

Clinical validation with physician assistance. In a clinical validation focused on occult scaphoid fractures, CNN assistance improved overall diagnostic sensitivity (from 0.72 without CNN to 0.87 with CNN assistance) and substantially improved interobserver agreement, while the statistically significant differences in time and confidence were judged unlikely to be clinically meaningful [6].

Radiologist-level performance and “marginal gains”. Another study reported an algorithm sensitivity of 72%, specificity of 93%, and an AUC of 0.88, with AI assistance improving interobserver agreement in some pairs and reducing reading time for some radiologists, yet without broad performance improvements for most radiologists. This illustrates a recurring pattern: the AI benefit is not uniform across levels of reader expertise [37].

Non-displaced femoral neck fractures. A focused deep-learning model for non-displaced femoral neck fractures using AP and lateral radiographs represents another “high-miss” application, aligned with ED safety needs where CT/MRI confirmation is costly or delayed [38].

#### 3.2.3. Fracture Characterization and Quantitative Measurements

Beyond binary fracture detection, several studies in the evidence base have extended to measurement automation, aiming to convert radiographs into objective metrics to support grading, follow-up, and treatment decisions.

A representative example demonstrates the development and validation of deep-learning-based quantification of thoracolumbar fracture features (e.g., compression rate, Cobb angle, sagittal index) from lateral radiographs, positioning AI as a “measurement co-pilot” rather than solely a detector [34].

This measurement direction is clinically important because it targets standardization and workflow speed—two prerequisites for integrating AI into trauma reporting and surgical planning.

#### 3.2.4. Decision Support in Trauma Pathways

A smaller portion of the trauma literature in the evidence base has moved beyond image interpretation into prognosis and decision support:

Risk prediction/nonunion: External validation of a nonunion risk score for tibial shaft fractures reflects the tradition of risk stratification in trauma; such tools serve as a natural bridge to modern ML but also highlight the need for robust external validation [39].

Outcome prediction in orthopedic trauma surgery: Feasibility work comparing ML and logistic regression for trauma outcomes demonstrates ongoing uncertainty about when ML materially outperforms standard methods inside heterogeneous trauma populations [40].

Secondary fracture risk after hip fracture: A CT-based deep-learning approach to predict subsequent fracture risk suggests a role for AI in secondary prevention pathways (fracture liaison services, anti-osteoporotic treatment triage) [33].

#### 3.2.5. Implementation and Workflow Impact

Implementation-focused studies in trauma consistently show that the largest performance gains are observed among less-experienced readers or non-specialists (e.g., ED physicians, residents). For experts (subspecialized MSK radiologists), AI often yields smaller or inconsistent improvements. This pattern is reinforced by reader-performance trauma work. Deep learning assistance closes the accuracy gap in fracture detection among clinician types [31]. The effect of deep convolutional neural networks on radiologists’ performance in the detection of hip fractures on digital pelvic radiographs [35].

### 3.3. Spine

#### 3.3.1. Deformity and Scoliosis: Automated Cobb Angle and Curve Quantification

Systematic evidence. A systematic review and meta-analysis in the evidence base consolidates deep-learning approaches for Cobb angle measurement, emphasizing reproducibility, comparison with human readers, and variation across methods and datasets [24].

High performance in standard settings. One algorithm-based approach reported a mean absolute error of ~1.97° and excellent reliability (ICC 0.981), supporting use as a measurement automation tool in routine clinical practice [51].

Performance limitations at the extremes. In a pediatric radiology context with severe and extreme curves, AI error increased and could be worse than that of residents overall (overall AI MAE 4.57°, and in extreme scoliosis, AI MAE 6.53° vs. residents 3.61°). This is a clinically important negative finding: the hardest cases may remain hardest for AI, and may require human monitoring or redesigned modeling strategies [53].

#### 3.3.2. Degenerative Spine: Imaging-Based Diagnosis and Surgical Candidate Stratification

Within the included evidence base, degenerative-spine AI is represented more heavily by perioperative outcome prediction and complication forecasting than by large-scale imaging-only diagnostic classification (e.g., disk herniation/stenosis grading). This distribution likely reflects both the complexity and the fluctuation of degenerative imaging phenotypes, as well as the availability of structured perioperative variables and outcomes that enable supervised learning.

#### 3.3.3. Predicting Outcomes After Spine Surgery

Spine outcome prediction studies in the evidence base illustrate several recurring points: (i) the importance of external validation, (ii) modest-to-good discrimination depending on endpoint, and (iii) limited benefit of ML over simpler models in some PROM settings.

PROM prediction with external validation: In lumbar spine surgery, models showed AUCs of ~0.63–0.72, explained variances of ~16–28%, and, notably, random forests did not outperform regression, underscoring that model complexity is not automatically beneficial [49].

Lumbar fusion outcomes: A machine learning model for lumbar spinal fusion reported a best-validation AUC around 0.81 (with other ML methods performing substantially worse in that dataset), indicating the sensitivity of results to algorithm choice and cohort specifics [54].

Cervical laminoplasty short-term outcomes: Large-cohort modeling reported AUROCs of ~0.83–0.86 for non-home discharge/unplanned reoperation/major complications, with additional outcomes (LOS/readmission) showing AUROCs of ~0.81–0.82 [48].

Revision prediction using labs/operative variables: Deep learning on standard labs and operative variables achieved an AUC of ~0.833 for short-term revision prediction in cervical spine surgery, suggesting potential for perioperative decision support but still calling for careful calibration and external validation [55].

#### 3.3.4. Spine Oncology/Metastasis: Bridge to MSK Oncology

A distinct cluster within “spine” addressed metastatic disease and functional outcomes: A machine-learning approach to predict postoperative neurological outcomes after metastatic spinal tumor surgery indicates the need for high-stakes decision support (expected neurological recovery vs. operative risk) [52].

#### 3.3.5. Follow-Up, Complication Surveillance, and Registry Integration

While explicit “model drift” and long-term surveillance frameworks proved less commonly operationalized across the spine papers, several studies used: temporal validation (PROM prediction); readmission modeling (including ML approaches); and text-based (NLP) extraction of operative complications, which together support the idea that spine AI is moving toward longitudinal and real-life data integration.

Predicting patient-reported outcomes following lumbar spine surgery: development and external validation of multivariable prediction models [49].

Development of machine learning and natural language processing algorithms for preoperative prediction and automated identification of intraoperative vascular injury in anterior lumbar spine surgery [50].

### 3.4. Arthroplasty

#### 3.4.1. Preoperative Imaging and Severity Assessment

Arthroplasty-related imaging AI in this evidence base is most visible through OA assessment automation and quantitative measurement:

A deep-learning-derived orthogonal minimum joint space width metric was proposed to improve radiographic assessment of OA severity together with progression, which is relevant to both surgical timing and standardizing radiographic endpoints in trials [67].

A more extensive review of deep learning methods in OA imaging contextualizes segmentation and morphometrics as building blocks for progression prediction and treatment stratification [25].

#### 3.4.2. Perioperative Risk Stratification

*Systematic review perspective.* Systematic review evidence in the evidence base indicates expanding use of ML for arthroplasty outcomes, with recurring limitations: retrospective designs, incomplete external validation, and variable reporting quality [14,26].

*Complication prediction: often moderate discrimination.* Some models achieved only moderate discrimination for major complications (AUC ~0.68) and performed poorly at predicting residual pain (AUC ~0.53), suggesting that not all endpoints are equally learnable from standard variables [59].

In a very large national cohort, multiple ML methods yielded AUCs of ~0.55–0.68 for major complications, illustrating the challenge of low-event-rate prediction even at scale [58].

*High-performing models exist for selected endpoints*. Some arthroplasty models performed strongly (e.g., DVT prediction after THA with AUC ~0.93 plus calibration/decision-curve utility) [57].

PJI prediction after THA showed good discrimination (AUC ~0.88) with explicit calibration metrics and top predictors highlighting process-of-care and comorbidity markers (e.g., LOS, ASA) [56].

#### 3.4.3. Implant Selection, Alignment, Navigation, and Robotics

Within the included evidence base, implant selection/navigation/robotics were presented more as review-level discussion than as a substantial body of dedicated validation studies. As a result, the evidence density here is lower than in fracture detection or complication prediction [1,26].

#### 3.4.4. Outcome Prediction: PROMs, Satisfaction, Revision, Survivorship

Discharge disposition and transferability. An external validation study of ML models for non-home discharge after revision TKA indicated stable performance spanning internal/external validation (AUC range 0.77–0.79), supporting generalizability for this specific endpoint [60].

Complications + operative duration. A model predicting complications in primary TKA reported high overall accuracy but low sensitivity (a classic imbalance issue), whereas predicting irregular surgery duration achieved a higher AUC [61].

Sleep disturbance as a patient-centered outcome. One study explicitly targeted postoperative sleep disturbance after TKA (a wider view of “outcomes” beyond surgical complications), illustrating the expanding perimeter of AI targets in arthroplasty [62].

#### 3.4.5. Postoperative Follow-Up and Surveillance

Within the included literature, postoperative surveillance evidence is primarily represented by short-term prediction of complications (e.g., readmission, infection, and DVT) and discharge disposition, rather than long-horizon detection of implant loosening or wear.

Representative examples include national-database models for predicting periprosthetic joint infection after primary total hip arthroplasty [56] and national-scale models identifying patients at high risk of deep vein thrombosis following primary total hip arthroplasty [57].

### 3.5. Sports Medicine and Soft-Tissue Orthopedics

#### 3.5.1. Knee: Meniscus and Ligament Injury Detection (MRI-Focused)

Meniscus tears. A systematic review/meta-analysis found that AI applied to MRI reliably detects meniscal tears but is less robust for the exact localization of tears. Reported pooled performance: AUC 0.939 (presence) vs. 0.905 (location), with pooled sensitivity/specificity roughly in the 0.87–0.89 range for tear identification [11].

ACL tears. Multi-continental external validation reported an AUC of 0.939, with sensitivity ~87% and specificity ~91%, strengthening the feasibility of robust ACL tear detection when external validation is explicitly designed [65].

#### 3.5.2. Cartilage and Osteoarthritis Biomarkers at the Sports–Early Degeneration Interface

Within the included literature, studies positioned at the intersection of sports medicine and early degeneration focused less on single-time-point diagnosis and more on quantitative imaging biomarkers of cartilage health. In this context, automated segmentation and morphometric quantification (e.g., cartilage thickness, volume, and subregional morphology) were treated as enabling steps, because reliable OA monitoring, progression assessment, and patient stratification require reproducible measurements rather than purely qualitative interpretation. Representative examples included deep learning–based knee MRI segmentation for fully automated subregional cartilage morphometry using Osteoarthritis Initiative data [10], and broader syntheses describing how deep learning supports OA imaging through segmentation-driven quantification and downstream prediction tasks [25].

#### 3.5.3. Shoulder: Rotator Cuff Tears and Muscle Quality

Rotator cuff tear screening. Multi-plane MRI screening showed best performance when all planes were used (reported AUC ~0.94, with very high sensitivity and accuracy in that configuration) [66].

Fatty infiltration quantification. “Trustworthy” deep learning approaches were targeted at clinically meaningful muscle-quality endpoints (fatty infiltration), achieving strong classification performance and high agreement with expert standards (ICC ~0.91, as cited in the study) [68].

Radiograph-based rule-out strategy. A deep learning approach to shoulder radiographs was proposed as a rule-out tool for rotator cuff tears (sensitivity ~97.3%, NPV ~96.6%), pointing to a potential gatekeeping role in pathways where MRI access is limited [69].

Synthesis/limitations. A focused review of shoulder MRI AI in the evidence base highlighted variable study quality and limited external validation in some studies, supporting the significance of rigorous multi-site testing before deployment [13].

#### 3.5.4. Rehabilitation and Return-to-Activity Follow-Up

Within the included evidence base, rehab/return-to-activity prediction was less densely represented than imaging diagnosis and surgical risk prediction. However, the direction of travel is implied by the increasing focus on PROM prediction (spine), postoperative complication and discharge modeling (arthroplasty), and the emergence of generative AI/LLM discussions as workflow tools.

A future-oriented synthesis on generative AI provides context for how documentation, education, and patient communication may become AI-augmented components of follow-up care: Integrating generative artificial intelligence into orthopedics: A review of opportunities, challenges, and future directions [20].

### 3.6. Musculoskeletal Oncology

#### 3.6.1. Tumor Detection and Differential Diagnosis

In the oncology subset, radiomics and ML/DL were the dominant paradigms. Included studies frequently involved small cohorts and heterogeneous imaging protocols, with variable adherence to reporting standards—factors that can limit generalizability despite high internal AUCs. A systematic review applying formal quality frameworks showed low average radiomics quality and incomplete adherence to reporting guidelines, underscoring why “excellent AUC” is not equivalent to deployability [23].

#### 3.6.2. Predicting Neoadjuvant Chemotherapy Response (Osteosarcoma-Focused)

Meta-analytic performance. A systematic review/meta-analysis reported pooled metrics for NAC response prediction in osteosarcoma: training cohorts AUC 0.93, sensitivity 0.89, specificity 0.85; validation cohorts AUC 0.87, sensitivity 0.81, specificity 0.82. Clinical-combined models tended to outperform radiomics-only models [22].

Single-study examples. Multiple individual studies have developed radiomics/nomogram models for chemotherapy response, frequently emphasizing combined models and reporting AUCs in the moderate-to-good range. For example, a clinical-radiomics nomogram reported an AUC of ~0.793 and an accuracy of ~79% in a test dataset [16].

#### 3.6.3. Metastasis and Survival Prediction

Pulmonary metastasis prediction. CT-radiomics-based ML was used to predict pulmonary metastasis risk, with one report noting test accuracy ~73% and AUC ~0.79 in the test set—promising but still within the “needs broad validation” category given typical cohort sizes and imaging heterogeneity [72].

Outcome stratification (pediatric OS). MRI-based radiomics/ML for pediatric osteosarcoma outcome stratification reported wide AUC ranges across outcomes and datasets, suggesting both potential and a risk of instability depending on endpoint definition and cohort shift [73].

Metastatic spine tumor outcomes (bridge). ML prediction of neurological outcomes after metastatic spinal tumor surgery further stresses oncology’s need for decision support in high-risk operative settings [52].

#### 3.6.4. Surgical Planning and Post-Treatment Surveillance

Across the included oncology literature, emphasis was placed on response prediction and metastasis risk/outcome stratification rather than on validated AI tools for margin planning or standalone post-treatment imaging surveillance. This pattern suggests a gap for future work: impact studies evaluating whether radiomics/ML changes surgical decision-making or surveillance strategies.

### 3.7. Additional Subspecialties Represented in the Literature

#### 3.7.1. Pediatric Orthopedics

Pediatric work in the evidence base illustrates both diagnostic assistance and more novel tasks (fracture dating):

Resident performance augmentation: AI access improved AUC for residents in pediatric/young-adult upper-extremity fracture detection (radiology residents: AUC 0.768→0.876; pediatric residents: AUC 0.706→0.844), with little improvement for subspecialized attendings [32].

Fracture dating (forensic/child protection relevance): Deep learning estimated infant clavicle fracture age with MAE ~4.2 days and high ICC (~0.919), positioning AI as a “virtual consultant” for a difficult, experience-dependent interpretation task [41].

#### 3.7.2. Hand and Upper Extremity

The scaphoid fracture literature (covered in Trauma 3.2.2) is effectively “hand and wrist AI,” and this evidence base includes: pipeline model development [5]; clinical validation with physician assistance [6]; and radiologist-level performance and detailed reader benefits [37].

#### 3.7.3. Foot and Ankle

The foot/ankle is represented via high-performing radiographic fracture detection: Detection of ankle fractures using deep learning algorithms [36].

### 3.8. Cross-Cutting Enablers (Across All Subspecialties)

#### 3.8.1. NLP for Orthopedics: Notes, Reports, Registries, Readmission Prediction

NLP appears in two clinically meaningful ways in this evidence base:

- **Readmission prediction and risk modeling:** Highlighted by systematic review and meta-analysis synthesis [15].
- **Automated identification of intraoperative complications from text:** For example, in anterior lumbar spine surgery, an NLP algorithm achieved an AUC-ROC of ~0.92 on temporal validation for detecting intraoperative vascular injury from operative narratives, demonstrating that text pipelines can achieve high performance for rare but critical events [50].

#### 3.8.2. Forward-Looking Commentary: LLMs and Generative AI in Orthopedics

While the core of this review focuses on empirically validated predictive and perceptual AI, generative AI and Large Language Models (LLMs) represent a rapidly emerging, albeit currently speculative, frontier in orthopedics.

The evidence base includes an explicit review of generative AI opportunities and governance concerns in orthopedics, highlighting emerging use cases (e.g., documentation, education, synthetic data) while highlighting safety risks (e.g., hallucinations, accountability, privacy), integrating generative artificial intelligence into orthopedics: A review of opportunities, challenges, and future directions [20]. Also, an orthopedic AI narrative review provides field-level context and terminology harmonization (ML vs. DL vs. clinical deployment issues) [19].

#### 3.8.3. Validation, Generalizability, and Implementation Maturity

Three recurrent “maturity markers” were apparent across subspecialties:

- **External/temporal validation:** Improves credibility yet remains not universally applied [49,65].
- **Reader/impact studies:** Essential to translate “accuracy” into “clinical benefit,” often showing the strongest gains in less-experienced clinicians [31,32].
- **Robustness testing:** Threats such as confounding and dataset bias can invalidate clinical assumptions if not explicitly tested [21].

## 4. Discussion

### 4.1. Answering the Title Question: “Who’s the Surgeon Now?”

Across this evidence-based review, the most defensible answer is that AI is not becoming “the surgeon.” Instead, it is becoming a family of high-throughput clinical instruments that can automate narrowly bounded perceptual tasks, standardize quantification, and generate probabilistic risk estimates—while the human surgeon remains the accountable decision-maker and the physical operator of invasive care. This framing is consistent across broad orthopedic syntheses, which repeatedly emphasize augmentation rather than replacement, largely because orthopedic practice couples interpretation (imaging + exam) with procedural execution, intraoperative adaptation, and longitudinal responsibility [1,2,27,28].

A practical way to move from rhetoric (“replace vs. augment”) to implementation is to decompose orthopedic work into four coupled layers—each with a different automation ceiling and a different safety profile:

**Perception/recognition (highly automatable, high immediate ROI):** Fracture detection on radiographs and common MRI classification tasks are the clearest near-term targets. The strongest translational studies in this review—multi-site fracture detectors and reader-assistance designs—support triage and second-reader workflows as the realistic deployment model, rather than autonomy [4,7,8,29,30,31].

**Quantification/standardization (highly automatable, clinically enabling):** Many effective orthopedic AI applications focus not on decision-making, but on converting images into repeatable measurements that are subject to human variability (such as Cobb angle, fracture morphology metrics, joint space width, and cartilage morphometrics). This approach offers significant value, as standardized quantification enables scalable monitoring, registry development, and consistent treatment-response endpoints [10,24,25,34,51,67].

**Prediction (partly automatable, but clinically fragile unless carefully validated):** Risk prediction (DVT, PJI, discharge disposition, readmission, PROMs) can be clinically useful *if* it is well-calibrated, externally validated, and operationalized with clear decision thresholds. However, this review shows substantial heterogeneity across endpoints and datasets, and systematic reviews warn that model sophistication does not consistently translate into improved clinical utility [14,15,49,56,57,58,59,60,63].

**Prescription, values, and accountability (fundamentally human-led):** Deciding what should be done (operative vs. nonoperative, timing, implant strategy, balancing competing risks, matching with patient goals) requires normative judgment in uncertain conditions. The evidence supports decision support and risk communication, not autonomous recommendations. This is precisely why “replacement” is the wrong benchmark: orthopedic care is a socio-technical workflow with moral and medico-legal accountability anchored in the clinician.

Among these layers, a “co-surgeon model” best corresponds to the evidence: AI for triage, quantification, and prediction, embedded in workflows with explicit human-in-the-loop checkpoints and clear accountability. The most translation-relevant studies in this review—reader-assistance designs—show that AI can narrow expertise gaps and improve safety, especially among trainees and non-specialists, while leaving final responsibility unchanged [31,32,37].

Finally, generative AI and LLMs intensify the “replacement” narrative, but this review views them primarily as workflow accelerators (documentation, education, summarization, planning support) that still require supervision, as confident fabrication (“hallucination”) is a known risk. In practical orthopedic terms, generative AI may become a productivity layer—not an autonomous clinical decision-maker—unless it is constrained under robust governance and source-grounding [20].

### 4.2. Where AI Is Strong Today vs. Where It Is Still Fragile

**Where AI is strongest today (based on this evidence base):**

(A) **Imaging detection and triage, especially on radiographs**

Trauma radiographs remain the most mature “high-volume, high-cost-of-miss” target. Multi-site and multi-anatomic fracture detection systems indicate strong overall discrimination, and reader-assistance studies show tangible workflow-relevant benefits (augmented sensitivity/specificity and reduced inter-reader variability), supporting ED triage and second-read paradigms. Hip fracture detection is a particularly compelling archetype because of its time-critical nature and well-defined downstream consequences [4,7,8,29,30,31,36,42].

(B) **Segmentation and measurement automation (quantitative imaging as a “clinical product”)**

Across OA imaging and spine deformity workflows, the deliverable is often not a diagnosis but a metric—cartilage morphometry, curve quantification, fracture morphometrics, or joint space width. This is an essential distinction: measurement automation can add value even when it does not “outperform” experts, because it boosts scalability, reproducibility, and downstream analytics (registries, trials, longitudinal monitoring) [10,24,25,34,51,53,67,70].

(C) **Selected perioperative risk models with more evident “actionability.”**

The strongest candidates for clinical use are risk models where (i) the endpoint is sufficiently learnable, (ii) calibration/utility framing is explicit, and (iii) the predicted risk links to an actionable pathway (e.g., intensified thromboprophylaxis pathways, infection surveillance, discharge planning). Arthroplasty provides examples at both ends of the spectrum: DVT/PJI prediction can be strong in big datasets, while prediction of residual pain or rare complications can remain modest and clinically ambiguous without a careful threshold strategy [14,56,57,58,59,60,61,62,64].

**Where AI remains fragile and why this matters clinically:**

(A) **“Hard-case” perception tasks: occult, subtle, localized, or severity-extreme cases**

Scaphoid fracture work shows a repeatable pattern: strong performance in obvious cases, lower performance in occult cases, and reader-dependent benefit when AI is integrated into clinical reading. Similarly, in meniscal tears, meta-analytic evidence suggests that AI is more reliable at detecting tear presence than at localizing the tear, even though localization often guides surgical strategy. Severe/extreme deformity further strains measurement algorithms, demonstrating that the decision-critical tails of the distribution may remain the hardest to automate [5,6,11,37,38,43,53].

(B) **Prediction in multi-morbidity contexts and low-base-rate endpoints**

When events are rare (e.g., major complications) or multifactorial (e.g., pain, functional impairment), predictive discrimination can be modest, and clinical utility depends heavily on calibration and the downstream decision policy. Several arthroplasty and spine papers show that more complex algorithms do not dependably outperform regression, particularly for PROM prediction; this is a critical point for shared decision-making tools, where miscalibration can mislead both surgeons and patients [14,48,49,54,58,59].

(C) **Musculoskeletal oncology radiomics: promising but methodologically vulnerable**

Oncology studies often report strong internal performance, but consistent evidence shows heterogeneous quality, incomplete external validation, and variable adherence to reporting standards—conditions that increase overfitting plus limit transportability. Meta-analytic signals for predicting osteosarcoma response are encouraging, but the translational threshold is higher because decisions are high-stakes and datasets are typically smaller [16,17,22,23,73].

**Core interpretive point:** the evidence base repeatedly shows that performance is not equal to clinical utility. For decision-influencing tools (risk models, pathway recommendations), discrimination alone is insufficient; calibration, decision-curve utility, external validation, and impact evaluation are the true translation filter [14,49,56,57].

### 4.3. Generalizability and Dataset Shift

A central translational gap across the evidence base is that model effectiveness is often distribution-dependent. Even when internal validation is strong, real deployment requires robustness across:

- Institutions (academic vs. community; referral patterns; prevalence shifts)
- Devices/vendors (radiography equipment, MRI sequences, reconstruction kernels)
- Protocol and positioning variability (projection differences, artifacts, implants)
- Severity and case-mix shift (extremes of deformity, pediatric cohorts, atypical phenotypes)

The clearest signals of maturation are studies that are explicitly designed for external validation. Multi-continental external validation in ACL tear detection exemplifies both feasibility and the practical requirement for adaptation when moving between continents and protocols [65].

Generalizability is also endpoint-specific in arthroplasty and spine: discharge disposition models can generalize reasonably when the endpoint is well-defined, and the data-generating process is stable, whereas PROM prediction and multi-morbidity outcomes commonly exhibit more modest, context-dependent transportability [49,60].

In fracture detection, even multi-site systems show anatomic-region variability, which is a practical warning that “one model for all MSK radiographs” can still underperform in certain subdomains (e.g., foot/ankle), notably under distribution shift [30,36].

Dataset shift can also be a clinical severity shift, not only a scanner shift. Scoliosis measurement in severe/extreme curves demonstrates that the cases surgeons care most about may be precisely where automation becomes least reliable, underscoring the requirement for explicit “tail-of-distribution” evaluation [24,53].

Finally, generalizability is not a one-time achievement: it requires post-deployment monitoring because medical workflows evolve, imaging procedures change, and populations shift (concept drift). Radiology-facing orthopedic AI discussions increasingly highlight PACS integration and post-market monitoring as prerequisites for sustained safety and value [3].

### 4.4. Bias, Confounding, and Hidden Shortcuts

The most important cautionary signal in the evidence base is that apparent performance can be achieved through shortcuts that are not clinically meaningful. The canonical example is hip fracture confounding: deep learning can appear to “predict hip fracture,” yet exploit confounding patient and healthcare process variables (or proxies embedded in images), with performance collapsing when confounders are balanced. This directly questions the assumption that a high AUC equates to “seeing the fracture” [21].

- This risk generalizes across orthopedics because shortcut channels are everywhere:
- Projection artifacts correlated with immobilization, pain severity, or workflow urgency
- Hardware/implants as proxies for prior disease or surgery
- Institutional signatures (vendor-specific image traits; annotation conventions)
- Label leakage (labels derived from downstream CT/MRI or surgical outcomes embedded in text)
- Spectrum bias (too many obvious cases; too few subtle, pediatric, or atypical cases)

Fairness is not hypothetical. Real-world evaluations that examine demographic factors (including ethnicity) underscore the need to measure subgroup performance rather than assume it—especially for triage tools and models that influence access to imaging, surgery, or discharge resources [8].

Bias risk is amplified in small-data domains such as musculoskeletal oncology radiomics, where systematic quality assessment reveals recurrent deficits in reproducibility safeguards (e.g., reporting completeness, external validation, failure analysis). These deficits are exactly where hidden bias often resides [22,23].

Discussion implication: for orthopedic AI—particularly tools affecting triage, operative indication, or discharge—bias/confounding assessment should be treated as a core validation axis alongside discrimination, with explicit subgroup reporting and sensitivity analyses [14,19,21].

### 4.5. Ground Truth Problems in Orthopedic AI

Orthopedic AI is unusually vulnerable to ground-truth ambiguity because “truth” can be defined at multiple levels:

- **Imaging truth:** Radiographic appearance.
- **Reference imaging truth:** CT or MRI confirmation.
- **Operative truth:** Arthroscopy or intraoperative findings.
- **Pathologic truth:** Tumor histology, necrosis, and margins.
- **Longitudinal truth:** Patient-reported outcome measures (PROMs), revision, recurrence, and survivorship.

The scaphoid fracture literature demonstrates best practice: occult fractures are defined using CT/MRI follow-up rather than radiographic labels alone, thereby improving label fidelity in a decision-critical workflow. But it also emphasizes a key tension: better reference standards can introduce label leakage if they encode downstream testing patterns rather than the signal intended for triage at first presentation [5,6,37].

In sports medicine, ground truth heterogeneity is often even more consequential. The clinically definitive diagnosis for meniscal/ACL pathology is often made via arthroscopy, yet many datasets rely on imaging interpretations. Meta-analytic evidence of weaker performance for tear location may partly reflect ground-truth granularity and inter-reader variability. Multimodal models anchored to arthroscopy-defined outcomes illustrate one pathway to higher-fidelity endpoints, but they also call for careful design to avoid leakage and spectrum bias [11,65,71].

In oncology, “truth” is usually biology and outcomes rather than imaging appearance: histologic necrosis after chemotherapy, metastasis, and survival. This increases the translation bar. Systematic evidence and quality assessments underscore that repeatability and reporting discipline (reference-standard clarity, external validation, harmonization) are not “administrative details”—they determine whether the model can be trusted [16,17,22,23,73].

Even apparently “simple” labels can be unstable. Cobb angle shows meaningful inter-reader variability, and surgical AIS cohorts may reflect a different measurement distribution than general clinics—again pointing out that ground truth is not a single value but rather a distribution [24,51,53].

Finally, ground truth in NLP-based orthopedics often depends on coding, registries, and the structure of operative narratives. High ability in detecting rare events (e.g., vascular injury from operative notes) is promising, but “truth” depends on record quality and a consistent narrative structure—making governance and standardization essential [15,50].

Discussion implication: orthopedic AI studies should explicitly declare a ground-truth hierarchy and justify why that truth is appropriate for the intended action (triage vs. operative planning vs. prognosis), ideally with adjudication strategies and sensitivity analyses when “truth” is uncertain or multi-source.

### 4.6. Explainability, Trust, and Human Factors

The evidence base indicates that trust is earned through workflows, not through ROC curves. Three actionable themes recur:

(1) **Explainability is often implemented as visualization, but visualization is not understanding.**

Heatmaps and saliency maps can increase face validity (e.g., fracture localization), but they can also be misleading if the model relies on confounders or if saliency is unstable. The hip fracture confounding warning is a key counterweight: a model can “look explainable” while being driven by non-fracture correlates [5,7,21].

(2) **Uncertainty communication may matter more than post hoc explanations.**

Clinicians need to know when *not* to trust outputs: borderline cases, artifacts, rare variants, severe deformity, postoperative hardware, and pediatric growth plates. “Trustworthy AI” framings that emphasize robustness, reliability, and clinically meaningful endpoints are more aligned with orthopedic safety than purely interpretability-focused narratives [53,68].

(3) **Human factors: AI benefit is user- and context-specific.**

Across reader-assistance studies, gains are often larger in trainees/non-specialists than in subspecialty experts. This carries practical implications for deployment: the same tool may be safety-critical in the ED at night but offer marginal benefit in a tertiary MSK radiology service. Even when the accuracy changes among experts are small, reductions in time-to-read and increased agreement may still be operationally meaningful [31,32,37,43].

In addition, orthopedic teams should anticipate classic human-AI interaction risks: automation bias (over-trusting the model), alert fatigue (ignoring frequent prompts), and potential deskilling if training settings substitute AI for cognitive practice without guardrails. Reviews in MSK radiology emphasize that integration quality (how alerts appear in PACS, how outputs are displayed, how overrides are audited) can be as important as algorithm choice [3].

### 4.7. Regulatory, Ethical, and Medico-Legal Considerations

As orthopedic AI transitions from research to clinical implementation, regulatory and governance frameworks dictate its real-world deployability. While the FDA and European agencies (CE mark) have cleared an increasing number of musculoskeletal AI systems—primarily for radiological triage and fracture detection—comprehensive real-world deployment data remains sparse. Regulatory clearance indicates algorithmic safety under specific conditions but does not guarantee clinical utility or cost-effectiveness in diverse, unselected populations. Furthermore, clinical translation introduces complex medico-legal and ethical challenges that require structured analysis:

- **Liability in Diagnostic Errors:** The integration of AI shifts traditional liability paradigms. If an AI system misses a subtle fracture (false negative) and the clinician relies on this assessment, the apportionment of liability between the software developer, the healthcare institution, and the individual surgeon remains legally ambiguous. Conversely, ignoring an AI-generated alert (false positive) that later proves correct exposes the clinician to distinct malpractice risks.
- **Informed Consent:** For algorithmic risk prediction (e.g., perioperative complication forecasting), the role of AI must be transparently communicated to the patient. Utilizing predictive models for shared decision-making requires informed consent regarding how the algorithm weighs variables and the inherent uncertainty of its probabilistic outputs.
- **Demographic Bias and Fairness:** Predictive models and imaging algorithms trained on homogeneous historical datasets risk perpetuating demographic bias. If a model systematically underperforms for underrepresented ethnicities or specific socioeconomic groups, it can exacerbate existing healthcare disparities, particularly when used for triage or resource allocation.

Consequently, adoption must be paired with explicit institutional governance. This includes active post-market surveillance to monitor model drift, structured patient-consent protocols for AI-assisted surgical planning, and continuous audits for demographic fairness [3,19,20,21,23].

### 4.8. Economic and Workflow Considerations

From an operational standpoint, the most credible near-term ROI in this evidence base is process efficiency and mistake reduction in high-volume imaging pathways—particularly emergency radiographs.

- Multi-site fracture detection performance supports worklist prioritization and earlier detection of time-critical injuries, with the largest downstream value plausibly in ED throughput and reduced diagnostic delay [30].
- Reader-assistance studies are economically relevant because they quantify benefit in human workflow terms (e.g., improved sensitivity/specificity, reduced variability), which maps to quality/safety metrics [31,32].
- Efficiency gains can be incremental but meaningful: reduced reading time and improved agreement even when expert accuracy is unchanged [37].

However, economic reality is not only about “AI saves time.” The evidence base implies substantial hidden costs:

- Integration costs (PACS/EHR, UI/UX, alert routing)
- Maintenance costs (model updates, drift monitoring, revalidation)
- Governance costs (privacy/security, auditing, oversight committees)
- Training costs (clinicians must learn appropriate reliance and override behavior)

Radiology-focused syntheses emphasize that integration and monitoring are prerequisites for value realization, not optional extras [3].

On the prediction side, economic value depends on whether the risk score changes an actionable pathway. Readmission prediction and arthroplasty outcome modeling are often motivated by value-based care, although systematic reviews indicate that external validation and consistent demonstration of utility are not universal, limiting direct economic translation [14,15].

Discussion implication: cost-effectiveness is most plausible where (i) case volume is high, (ii) missed diagnoses are costly, and (iii) AI improves triage or reduces delays; it is less plausible for low-volume, highly heterogeneous endpoints with modest predictive performance and unclear actionability [4,14,30,31].

### 4.9. Future Directions and Research Agenda

This review suggests that the next phase of orthopedic AI will be defined less by incremental AUROC gains and more by systems-level integration and evidence quality. Several concrete research directions emerge:

(1) **Multimodal and “foundation-like” orthopedic AI**

Orthopedic decisions are inherently multimodal (imaging + labs + comorbidities + text + PROMs). The evidence base already includes multimodal strategies anchored to high-fidelity endpoints (e.g., arthroscopy-defined pathology), implying that the next generation of models will progressively integrate multiple data types rather than “single-modality” classifiers [19,71].

(2) **Prospective, impact-focused trials (beyond reader studies)**

A major gap remains in prospective evidence on patient- and system-level outcomes (time to treatment, reduction in complications, return visits, PROM trajectories). Reader studies are a strong transitional design—but not the endpoint for clinical integration [14,31].

(3) **Better reporting standards and reproducibility safeguards**

Oncology radiomics is the cautionary domain: meta-analytic promise may coexist with weak reporting and limited external validation. Systematic quality assessments explicitly call for more rigorous reporting to support reproducible translation [22,23].

(4) **3D/volumetric AI for complex fractures and planning**

Moving from “fracture yes/no” to structured classification and surgical planning support is a clinically meaningful frontier. CT-based 3D segmentation and classification assistance (e.g., tibial plateau fractures) can directly support training, planning, and standardization [44].

(5) **Secondary prevention and longitudinal risk pathways**

CT-based deep learning for subsequent fracture risk after hip fracture suggests a role for AI in fracture liaison services and secondary prevention—i.e., not only diagnosing the index event but triaging preventive interventions after it [33].

(6) **Expanding past conventional imaging**

The evidence base includes exploratory work using nontraditional modalities (e.g., infrared thermal imaging in pediatric wrist fractures). These are early-stage, but they point to future “sensor fusion” and non-radiographic triage tools [45].

(7) **Democratization (AutoML/no-code) with strong governance**

No-code ML may reduce barriers for QI and prototyping, but it increases the risk of under-validated tools being used beyond their safe scope. This makes governance, validation, and monitoring even more critical [46].

(8) **NLP-driven surveillance and documentation pipelines**

Text pipelines can allow scalable detection of rare complications and support real-world safety monitoring—if documentation is consistent and governance is strong. This direction is particularly important for post-market monitoring and quality programs [3,15,50].

### 4.10. Practical Recommendations for Clinicians and Researchers

Below is a more detailed, workflow-first **implementation checklist** distilled from recurring strengths and shortcomings in the evidence base. It is designed to separate “research-grade” models from clinically deployable tools.

A. **Before adoption (problem selection and evidence threshold)**
  1. **Define the clinical decision and failure cost:** Is the tool a triage flag, a quantitative measurement engine, or a decision-influencing risk model? Prioritize high-volume, high-cost-of-miss workflows (e.g., ED fracture triage) [4,30].
  2. **Demand external validation in comparable settings:** Multi-center, multi-vendor, and severity-spectrum validation should be considered minimum evidence [47,60,65].
  3. **Require evidence of clinical impact, not only accuracy:** Prefer reader/impact evaluations where possible; they are closer to deployability than retrospective AUC alone [31,32].
B. **Validation and safety (bias, truth, and calibration)**
  1. **Actively test for shortcut learning and confounding:** Require subgroup performance reporting, balanced test sets where feasible, and explicit failure analysis [8,21].
  2. **Specify ground truth and provenance:** Prefer CT/MRI confirmation for occult injuries; arthroscopy for meniscus/ACL when clinically indicated; and pathology endpoints in oncology, where appropriate [6,22,71].
  3. **For risk models, prioritize calibration and utility over AUC:** Require calibration reporting, decision thresholds tied to actions, and (ideally) decision-curve analyses [49,56,57].
C. **Deployment (human factors and monitoring)**
  1. **Test in the actual user population:** Benefits may concentrate in trainees or non-experts; evaluate by user group and setting (e.g., ED vs. elective clinic) [31,32].
  2. **Engineer the workflow and UI explicitly:** Decide how alerts enter the PACS/EHR, mitigate alert fatigue, and define how outputs are displayed (heatmaps, probabilities, uncertainty) [3].
  3. **Establish governance and post-deployment monitoring:** Ensure version control, audit trails, periodic revalidation, drift surveillance, and a clear escalation pathway for safety events [3].
  4. **Treat generative AI as assistive unless rigorously constrained and validated:** Use for documentation/education first; avoid ungrounded clinical decision support without strong governance [20].
  5. **For researchers:** adopt transparent reporting standards and reproducibility safeguards. Especially for radiomics and small-data oncology, failure analysis and external validation should be the default expectations [22,23].

In summary, based on the maturity of evidence across subspecialties in this review, the most realistic near- to mid-term outcome is a co-surgeon model. In this paradigm, AI functions as a rapid probabilistic assistant that standardizes detection, quantification, and selected risk predictions, while surgical decision-making, intraoperative adaptation, patient communication, and accountability remain fundamentally human-led [1,2,19,28].

## 5. Conclusions

Across the evidence-based literature, AI is not positioned to replace the orthopedic surgeon. Instead, it is emerging as a rapidly expanding set of high-throughput clinical instruments designed to assist with narrow, well-defined tasks—most notably imaging-driven fracture triage, automated anatomical measurement, and selected prognostic modeling. Orthopedics is moving toward a machine-augmented paradigm, where speed and standardization are added to human clinical reasoning.

However, current algorithmic performance claims are frequently fragile when exposed to real-world clinical complexity. The evidence base remains constrained by retrospective designs, a lack of prospective clinical impact studies, and vulnerabilities to dataset shift, label noise, and hidden confounding. Because of these methodological limitations, the concept of a “co-surgeon” model remains a forward-looking paradigm rather than a currently established standard of care. In the near- to mid-term, AI is best conceptualized as an assistive layer for triage and quantification, while the surgeon unequivocally retains the moral, medico-legal, and procedural accountability for patient outcomes.

The practical implication is that the next phase of AI in orthopedics should be judged less by incremental gains in discrimination metrics and more by clinical utility and governance: external and local validation in representative settings, explicit calibration and threshold strategy for actionable predictions, transparent ground-truth justification aligned to the clinical decision, bias and subgroup evaluation, human-factors design that supports correct use under pressure, and post-deployment monitoring for drift and safety. Generative AI may accelerate documentation, education, and communication, but it should remain assistive unless it is rigorously validated, source-grounded, and governed to prevent confident but incorrect outputs from contaminating care.

Ultimately, the question posed by this review—“*Who’s the surgeon now: human hands or machine minds?*”—has a clear answer supported by the literature: the surgeon remains human, but orthopedic practice is entering an era where the most effective and safest systems will increasingly be those that pair human accountability and procedural expertise with AI-enabled perception, quantification, and prediction. The strategic goal is not replacement; it is building a partnership that is demonstrably better than either alone.

## Figures and Tables

**Table 1:** Summary of AI Clinical Maturity and Validation by Orthopedic Subspecialty.

Subspecialty	Primary AI Tasks	Common Data Modality	Predominant Validation Stage	Included Original Studies (Refs.)	Key Translational Barriers
**Trauma and Fracture**	Detection, Triage, Automated Measurement	Radiographs, CT	Internal, Emerging External, Reader-Impact Studies	[5,6,7,8,21,30,31,32,33,34,35,36,37,38,39,40,41,42,43,44,45,46,47]	Confounding, spectrum bias (occult fractures), and anatomical variability
**Spine**	Curve quantification, PROM/Complication Prediction	Radiographs, Clinical Tabular	Internal, Temporal, limited External (selected endpoints)	[48,49,50,51,52,53,54,55]	Calibration failure at deformity extremes, modest PROM prediction
**Arthroplasty**	Perioperative Risk, Complication Prediction	Tabular, EHR, Radiographs	Internal, External (selected endpoints)	[56,57,58,59,60,61,62,63,64]	Low-event-rate prediction, dataset shift across institutions
**Sports Medicine**	Tear detection, Cartilage segmentation	MRI	Internal, External (multi-continental)	[9,10,65,66,67,68,69,70,71]	Ground truth heterogeneity (imaging vs. arthroscopy), tear localization
**Oncology**	Chemotherapy response, Metastasis prediction	MRI, CT (Radiomics)	Internal, Limited External	[16,17,72,73]	Small sample sizes, lack of standardized reporting, and overfitting risks

Note: The table above categorizes the 53 original AI development and validation studies included in this review. The remaining 20 references in our evidence base [1,2,3,4,11,12,13,14,15,18,19,20,22,23,24,25,26,27,28,29] comprise systematic reviews, meta-analyses, and narrative assessments. Because these papers synthesize prior literature rather than train or validate novel AI models, they do not possess a primary “Validation Stage” or “Data Modality” of their own and are therefore excluded from this model-centric summary. Validation stage reflects the predominant design across included studies within each subspecialty; exceptions are noted in-text.

## Data Availability

Not applicable (narrative review based on published literature).

## Abbreviations

3D	three-dimensional
ACL	anterior cruciate ligament
AI	artificial intelligence
AIS	adolescent idiopathic scoliosis
AP	anteroposterior
ASA	American Society of Anesthesiologists (physical status classification)
AUC	area under the curve
AUC-ROC	area under the receiver operating characteristic curve
AUROC	area under the receiver operating characteristic curve
AutoML	automated machine learning
CNN	convolutional neural network
CT	computed tomography
DL	deep learning
DVT	deep vein thrombosis
ED	emergency department
EHR	electronic health record
FDA	U.S. Food and Drug Administration
GAI	generative artificial intelligence
ICC	intraclass correlation coefficient
LLM	large language model
LOE	level of evidence
LOS	length of stay
MAE	mean absolute error
ML	machine learning
MRI	magnetic resonance imaging
MSK	musculoskeletal
NAC	neoadjuvant chemotherapy
NLP	natural language processing
NPV	negative predictive value
OA	osteoarthritis
OS	osteosarcoma
PACS	picture archiving and communication system
PJI	periprosthetic joint infection
PROM	patient-reported outcome measure
QI	quality improvement
RoB	risk of bias
ROC	receiver operating characteristic
ROI	return on investment
T2	T2-weighted
THA	total hip arthroplasty
TKA	total knee arthroplasty
UI	user interface
UX	user experience
